# Anaesthesia for ruptured ectopic pregnancy at district level

**DOI:** 10.4102/safp.v63i1.5304

**Published:** 2021-05-27

**Authors:** David G. Bishop, Simon P.D.P. le Roux

**Affiliations:** 1 Department of Anaesthetics, Critical Care and Pain Management, University of KwaZulu-Natal, Durban, South Africa; 2 Global Surgery Division, Department of Surgery, Faculty of Health Sciences, University of Cape Town, Cape Town, South Africa

**Keywords:** anaesthesia, resource-limited settings, emergency surgery, ectopic pregnancy

## Abstract

In South Africa, deaths as a result of ectopic pregnancies are increasing despite the overall improvements in maternal mortality. These deaths occur predominantly in district hospitals, with the final cause of death being hypovolaemic shock in almost all cases. In most cases, no anaesthesia was attempted despite the district hospitals having the clinical skills, equipment and infrastructure to provide a caesarean delivery service. It appears that there is a skills gap between the provision of anaesthesia for caesarean delivery and that of ruptured ectopic pregnancy. There is a growing recognition of the urgent need to prioritise the provision of emergency surgical care in rural settings. This should be viewed not as a luxury but as an absolute necessity. In this study, we aim to discuss the pathophysiology of a patient with a ruptured ectopic pregnancy briefly, outline district hospital requirements for safe surgery and then discuss a simple, safe method for the provision of anaesthesia in patients deemed too unstable to transfer to a referral facility.

## Introduction

The Lancet Commission on Global Surgery was established in recognition of the moral imperative to provide safe and accessible surgical care within global health initiatives.^1^ This commission recognised that ‘surgery and anaesthesia are integral, indivisible components of any properly functioning health system’.^1^ Yet data from the latest Saving Mothers report (2017–2019) suggest that deaths in early pregnancy are increasing despite a decreasing number of overall maternal deaths.^2^ In total, 323 deaths were reported during early pregnancy: 119 (37%) because of ectopic pregnancy and 204 (63%) because of complications of miscarriage.^2^ Deaths from ectopic pregnancies occur predominantly in district hospitals (37%), with the final cause of death being hypovolaemic shock in 81% of cases. In one-fifth of cases, no resuscitation was attempted, and anaesthesia was only administered in 38% of cases (indicating that almost two-thirds of cases had no attempt at surgical control of bleeding). The lack of appropriately trained doctors is highlighted as the most frequent administrative avoidable factor. This is despite these hospitals having the infrastructure, equipment and clinical skills to provide an obstetric caesarean delivery service: these are classified as avoidable deaths in approximately two-thirds of cases.^3^ It appears that there is a skills gap between the provision of anaesthesia for caesarean delivery and that of ruptured ectopic pregnancy. There is a growing recognition of the urgent need to improve the capacity to provide emergency surgical care in rural settings. This should be viewed not as a luxury but as an absolute necessity.^4^ In this article, we aim to discuss the pathophysiology of the patient with a ruptured ectopic pregnancy briefly, outline district hospital requirements for safe surgery and then discuss a simple, safe method for the provision of anaesthesia in patients deemed too unstable to transfer to a referral facility.

## Ectopic pregnancies: General principles

Ectopic pregnancy is a common, life-threatening condition that occurs when a fertilised ovum implants outside the uterine cavity.^5^ Whilst there are several different implantation sites and clinical presentations, this article will mainly deal with the acute ruptured ectopic pregnancy requiring surgery at a district hospital. It is important to recognise that very unstable patients may not survive transfer to a referral facility. These patients require urgent, on-site surgery.^2^ In addition, stable patients with a ruptured ectopic pregnancy may rapidly deteriorate and become haemodynamically unstable. It is, thus, always advisable to provide definitive, on-site management for patients with ruptured ectopic pregnancy. It is imperative that district hospitals develop the capacity to provide safe perioperative care to this group of patients.

There are two common errors in the management of ectopic pregnancies: missed diagnosis and failure to control the bleeding.^3^ It is critical that early diagnosis is made to enable management that prevents haemodynamic instability occurring. Diagnosis is based on clinical acumen and ultrasound where necessary. This aspect is covered in other publications.^3^ The initial clinical presentation is influenced by the amount of blood lost, which will alter the haemodynamic variables, ranging from haemodynamically stable patients to patients in haemorrhagic shock.^6^ Shock is initially a single component (hypovolaemic) but if not quickly managed may progress to multi-component shock (including both cardiogenic and vasodilatory elements).^7^ This may then be compounded by coagulopathy, which usually develops only after significant blood loss. Failure to perform a laparotomy occurs because there is either a failure to recognise hypovolaemia or because there is a reluctance to proceed to theatre in unstable patients. Recognising shocked patients may be simplified through by using the shock index (heart rate [HR]/systolic blood pressure [SBP]).^6^ Values > 1 suggest a significant shock. Table 1 summarises an outline of the assessment of stability based on the Advanced Trauma Life Support (ATLS) system.^6^

**TABLE 1:** Assessing haemodynamic stability.

Clinical parameter	Stable(blood loss < 15%)	Concerning(blood loss 15% – 30%)	Unstable(blood loss > 30%)
Heart rate	< 100	100–120	> 120
Systolic blood pressure (mmHg)	> 100	> 100	< 100
Level of consciousness	15	15	< 15
Respiratory rate	< 20	20–30	> 30

mmHg, millimetre of mercury.

Whilst this system is useful in assessing patients, the ATLS classification may underestimate the degree of blood loss in trauma patients.^8^ In patients with ruptured ectopic pregnancies, the physiological compensatory mechanisms of early pregnancy (increased cardiac output, increased plasma volume and an increased reliance on the sympathetic nervous system for maintenance of haemodynamic stability) may further mask significant bleeding.^5^ One must, therefore, always prepare for potential decompensation following induction of anaesthesia and loss of sympathetic compensatory mechanisms, even in patients who initially appear stable.

## Theatre and skills requirements

**FIGURE 1:**
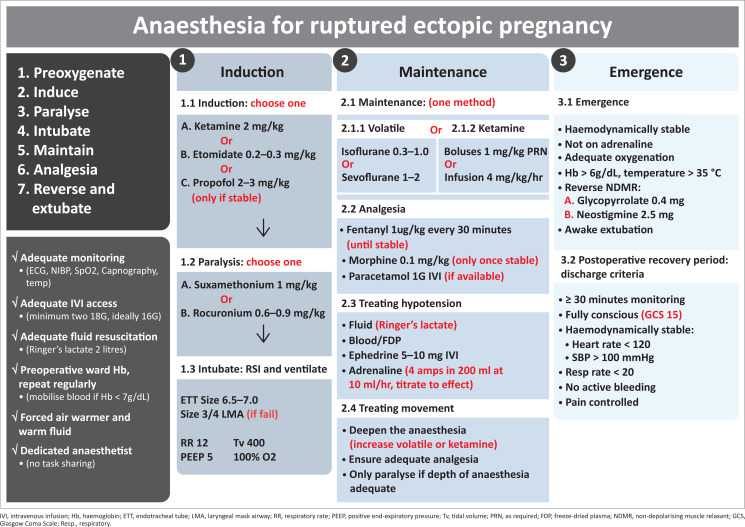
Anaesthesia for ruptured ectopic pregnancy.

Several international and national guidelines outline the basic requirements for a theatre service to provide a safe perioperative environment.^9^ The Essential Steps in the Management of Obstetric Emergencies (ESMOE) training programme has been shown to improve the knowledge and skill sets and incorporates a list of essential skills, equipment and a pre-anaesthetic checklist.^10^ These protocols should be laminated and displayed in theatre complexes. District hospitals should ideally appoint a *lead anaesthetist*. This is someone who has an interest in anaesthesia and takes the lead in theatre services. It is preferable, but not essential, that this person has attained a diploma in anaesthesia. As a minimum, clinicians should be able to perform fluid resuscitation and intubation and be able to set basic ventilator settings. With this skill set, there are simple anaesthetic techniques that will be immediately accessible (see Figure 1). When unstable patients are taken to theatre in a district hospital setting, the most senior staff available should be called to assist both in administering anaesthesia and in surgery.

## Principles of resuscitation

Resuscitation in the unstable patient is simply a bridge to going to theatre – surgical control is essential:

Being haemodynamically unstable is not a contraindication for anaesthesia in the case of a ruptured ectopic pregnancy; it is an urgent indication for anaesthesia to allow life-saving surgery and effective resuscitation.^3^

Crucially, haemodynamic stability prior to theatre is not the end point. Resuscitation occurs whilst transferring the patient to theatre, continues in theatre and concludes in the postoperative phase. Resuscitation can only be successful once the surgical control of the bleeding is achieved. Aim to keep the SBP > 90 mmHg and the HR < 120 beats per minute (bpm) if possible whilst preparing for theatre but do not aggressively target the normal blood pressure (SBP > 110 mmHg) in this phase, as this may increase the blood loss.^11^

### Haemostatic resuscitation

The best blood conservation strategy is early surgery. Delaying transfusion until surgical control is attained seems logical, unless the degree of maternal instability suggests the patient will not survive until surgery. Clinicians should be aware of local perioperative blood management guidelines.^12^ District hospitals should have stock of emergency blood on-site. In case of limited availability of blood, we suggest that this blood should be used pre-induction if the haemoglobin (Hb) is < 5 g/dL; be in theatre if the Hb is < 7 g/dL and be available from the fridge on short notice if the Hb is > 7 g/dL. The Hb can be measured with point-of-care devices (‘ward Hb’), and these should be routinely available in district hospitals. In the absence of a measured Hb, transfusion should be initiated based on clinical criteria: failure to respond to initial crystalloid fluid resuscitation (SBP < 90 mmHg) in combination with clinical pallor. Autotransfusion systems, such as cell savers, are invaluable ways to conserve blood and have proved life-saving in areas where blood is unavailable. Complex re-infusion systems are unlikely to be available at the district level, and low-technology methods should be considered for intraoperative autotransfusion. These low-technology systems are under-utilised in resource-limited settings.^13^

Freeze-dried plasma (FDP) is another useful product that should be stocked by district hospitals. It has a long shelf-life, can be stored at room temperature and is a useful colloid resuscitative fluid in the setting of massive haemorrhage, both for fluid resuscitation and for the replacement of clotting factors. It is unlikely that district hospitals will have access to blood products such as cryoprecipitate, but the administration of tranexamic acid (1 g intravenously) during massive haemorrhage is advisable to reduce fibrinolysis.

A suggested, step-wise fluid resuscitation strategy is outlined in the following:

- Establish large-bore intravenous infusion (IVI) access (minimum two 18 Gauge (G), ideally 14 G or 16 G) and check ward Hb.
- Give 1 L crystalloid rapidly: Ringer’s lactate (preferably) or normal saline.
- Target SBP > 90 mmHg, HR < 120 bpm.
- Repeat crystalloid (1 L) or colloid (e.g. Voluven 500 mL) if haemodynamic targets are not attained.
- Repeat ward Hb regularly (every 30–60 min until bleeding is controlled).
- If Hb < 7: give FDP (2–4) plus fetch blood (administer blood if Hb < 5 pre-induction).
- If SBP < 90 mmHg after fluids, initiate adrenaline infusion and expedite surgery.

### Inotropic support

Pharmacological support of the blood pressure may be required if SBP targets are not attained with fluid resuscitation. Ephedrine boluses (5 mg – 10 mg) may be initially employed, but tachyphylaxis quickly occurs.^5^ Phenylephrine boluses/infusion are less suited to hypovolaemic shock, as it is a pure vasoconstrictor (although it may raise cardiac output through redistribution of venous blood from the splanchnic circulation).^14^ Failure to respond to repeated ephedrine boluses suggests that adrenaline may be needed to support the blood pressure. It is better to start adrenaline early but always in association with fluid resuscitation. Consider adrenaline when SBP < 90 mmHg following fluid resuscitation. It is always preferable to give adrenaline on a dedicated line if possible. Depending on equipment available, there are several methods of giving adrenaline. Some basic options, based on the available equipment, are as follows:

- Volumetric infusion pump: Put 4 amps (ampoules) (4 mg) in 200 mL normal saline and run at 10 mL/h initially. Titrate rapidly upwards until SBP > 90 mmHg.
- Syringe driver: Put 1 amp (1 mg) in 50 mL normal saline and run at 10 mL/h. Titrate rapidly upwards until SBP > 90 mmHg.
- No syringe driver or volumetric infusion pump: Put 1 amp adrenaline (1 mg) in 1 L Ringer’s lactate and run freely. Titrate fluid rate to effect.

## Contextualised anaesthesia care

There are several approaches to anaesthesia in the unstable patients. A simplified approach is shown in Figure 1, which incorporates suggestions made in the Guidelines for Maternity Care in South Africa.^15^ Underlying this approach are several important anaesthetic principles outlined below.

### Principle 1: All anaesthesia is a titration

Unstable patients require far less anaesthetic agent than stable patients. Conventional doses may be dangerously high for unstable patients. Similarly, unstable patients who are responding to resuscitation may require higher doses once they begin to recover. Always start on the lower end of the dosage range and work upwards. Propofol is generally not advised in the unstable patient unless an experienced anaesthetist is available, as it may cause profound hypotension. It is preferable to use ketamine or etomidate in these circumstances, as shown in Figure 1.

### Principle 2: Unstable patients are at high risk of awareness

Awareness is an unavoidable risk of anaesthetising unstable patients: it is necessary to maintain a lighter plane of anaesthesia to prevent cardiovascular collapse. Once stability is achieved, it is important to deepen the anaesthetic, adequately address analgesia (with multimodal analgesia) and counsel patients postoperatively regarding awareness. Midazolam can be used to reduce the risk of awareness (2.5 mg – 5 mg), but its vasodilatory actions may make it unsuitable in the haemodynamically unstable patient.

### Principle 3: The goal of airway management is ventilation and oxygenation, not intubation

A major concern for occasional anaesthetists is the management of the airway during a general anaesthetic. Patients should be assumed to have a full stomach and, therefore, require a rapid-sequence induction, including cricoid pressure.^16^ Whilst it is possible to do a laparotomy under pure ketamine (with only facemask oxygen), this should not be advocated as a general rule. Skills to intubate patients and employ basic ventilation are essential at the district hospital level. If the clinician is unable to insert an endotracheal tube, mask ventilation should be attempted and, if possible, a supraglottic airway device (SGA) should be inserted.^16^ It is acceptable to do a laparotomy using an SGA if one failed to secure endotracheal intubation. The goal is to provide enough ventilation to adequately oxygenate the patient. The latest airway guidelines stress the importance of pre-oxygenation, apnoeic insufflation (keeping the facemask firmly applied with oxygen at high flow until intubation is possible) and even allow for bag-mask ventilation in patients with cricoid pressure whilst awaiting paralysis, using pressures < 20 cm/H_2_O.^16^

### Notes on ketamine

Ketamine can be administered as a sole anaesthetic agent. Thus, induction (2 mg/kg IVI) can be followed by maintenance (repeated boluses of 1 mg/kg PRN or infusion at 4 mg/kg per hour). To run a 4 mg/kg per hour infusion, simply put 200 mg ketamine in a 50-mL syringe with normal saline and run at the patient’s weight. It should be remembered that whilst ketamine provides good anaesthesia and analgesia, it is less effective in preventing the movement of the patient. Accordingly, many anaesthetists will combine this technique with muscle relaxation (vecuronium, cisatracurium or rocuronium) or with a low dose of volatile anaesthesia (e.g. end-tidal isoflurane 0.3–0.5). If muscle relaxation is employed, then patients must be reversed with neostigmine and glycopyrrolate at the end of the procedure, prior to extubation.

## Choosing an anaesthetic method

The anaesthetic methods suggested here are techniques for occasional anaesthetists where the goal is to reduce the chance of significant harm to the shocked patient: if a more comprehensive anaesthetic skill set is available, then alternative techniques are advised.^15^ Prior to conducting anaesthesia, it is important that the essential equipment list for anaesthesia is available, and that the pre-anaesthetic checklist has been conducted in accordance with the national ESMOE guidelines: these habits should be institutional norms.^10^ These are widely available standards, and the protocols should be laminated and placed on theatre walls. The use of the World Health Organization (WHO) surgical safety checklist is also strongly advised.^17^

The anaesthetic employed should be based on the stability of the patient and the skill set and equipment available. For stable patients with an unruptured ectopic pregnancy, it is acceptable to use spinal anaesthesia (hyperbaric bupivacaine 0.5%: 2.6 mL – 2.8 mL and add fentanyl 10 *μ*g if available).^5^ However, it is not acceptable to use spinal anaesthesia if there are concerns regarding haemodynamic stability (see Table 1), significant bleeding or a low Hb (< 9 g/dL). In this setting, use the algorithm in Figure 1 to choose a method of general anaesthesia. Remember that advice is available: call a referral centre for telephonic assistance from the anaesthetic department.

## Conclusion

It is vital that district hospitals develop the skills required to provide perioperative care for patients presenting with ruptured ectopic pregnancy. Using the key principles of adequate resuscitation and appropriate airway management, it is possible to provide safe anaesthesia and analgesia for this group of patients. Unstable ectopic pregnancies are responsible for a large number of avoidable maternal deaths, and key to combatting this problem is that district facilities develop the expertise to operate on-site. Unstable patients are unlikely to survive transfer. By following the principles outlined in this article, it should be possible to safely achieve surgical control in the majority of patients.
